# Common Polymorphisms in the *RGMa* Promoter Are Associated With Cerebrovascular Atherosclerosis Burden in Chinese Han Patients With Acute Ischemic Cerebrovascular Accident

**DOI:** 10.3389/fcvm.2021.743868

**Published:** 2021-10-15

**Authors:** Qingzhe Hu, Zhenlei Chen, Xiaofan Yuan, Shucheng Li, Rongrong Zhang, Xinyue Qin

**Affiliations:** Department of Neurology, The First Affiliated Hospital, Chongqing Medical University, Chongqing, China

**Keywords:** RGMa, atherosclerosis, ischemic stroke, functional polymorphism, vasoprotection

## Abstract

Repulsive guidance molecule a (RGMa) plays a vital role in the progression of numerous inflammatory diseases. However, whether it participates in atherosclerosis development is not known. Here, we explored the influence of RGMa in atherogenesis by investigating whether an association exists between functional polymorphisms in the *RGMa* promoter and cerebrovascular atherosclerosis burden (CAB) in Chinese Han patients diagnosed with acute ischemic cerebrovascular accident. To this end, we conducted a genetic association study on 201 patients with prior diagnoses of acute ischemic stroke or transient ischemic attack recruited from our hospital. After admission, we conducted three targeted single-nucleotide polymorphisms (SNPs) genotyping and evaluated CAB by computed tomography angiography. We used logistic regression modeling to analyze genetic associations. Functional polymorphism analysis indicated an independent association between the rs725458 T allele and increased CAB in patients with acute ischemic cerebrovascular accident [adjusted odds ratio (OR) = 1.66, 95% confidence interval (CI) = 1.01–2.74, *P* = 0.046]. In contrast, an association between the rs4778099 AA genotype and decreased CAB (adjusted OR = 0.10, 95% CI = 0.01–0.77, *P* = 0.027) was found. Our Gene Expression Omnibus analysis revealed lower *RGMa* levels in the atherosclerotic aortas and in the macrophages isolated from plaques than that in the normal aortas and macrophages from normal tissue, respectively. In conclusion, the relationship between *RGMa* and cerebrovascular atherosclerosis suggests that *RGMa* has a potential vasoprotective effect. The two identified functional SNPs (rs725458 and rs4778099) we identified in the *RGMa* promoter are associated with CAB in patients diagnosed with acute ischemic cerebrovascular accident. These findings offer a promising research direction for *RGMa*-related translational studies on atherosclerosis.

## Introduction

Atherosclerosis (AS), a common pathogenesis associated with intracerebral or extracerebral artery stenosis, can cause a series of ischemic cerebrovascular events in people. The medical burden of cerebral AS (CAB) is also associated with ischemic stroke recurrence, which affects stroke prognosis. AS is currently universally recognized as a chronic inflammatory change involving the participation of various cell types and cytokines, and it is hard to reverse. The pathophysiology of AS is still debated among scientists. Previous studies have shown that the neural guidance protein (NGP), a type of pluripotent molecule, affects neural guidance, cell apoptosis, and inflammatory regulation in various disease processes, and influences AS development (1).

Bone marrow-derived monocyte infiltration into the intima is considered a signature of AS development, whereas its emigration from the vessel walls enables AS remission (2). Numerous NGPs and/or their receptors can influence these processes. One well-studied NGP, netrin-1, is secreted by macrophages in plaques and exerts a pro-atherogenic effect by prohibiting macrophage emigration from vessel walls (3, 4). Nevertheless, it seems paradoxical that netrin-1, which is expressed by endothelial cells, can prevent AS by inhibiting inflammatory cytokine secretion and monocyte recruitment (5). Semaphorin 3E (Sema3E), a critical NGP in inflammation, is highly upregulated in macrophages from late-stage AS plaques (6). Sema3E inhibits macrophage emigration in a Plexin D1-dependent manner, which hampers the elimination of AS-related inflammation (7). In addition, vascular smooth muscle cell-derived Sema3E decreases macrophage migration and proliferation, thereby limiting neointimal formation (8). Therefore, it is believed that NGP exerts diverse functions in AS progression depending on its temporospatial distribution.

Repulsive guidance molecule a (RGMa), which is encoded by the *RGMa* gene (NCBI Entrez Gene: 56963), is a glycosylphosphatidylinositol-linked NGP with functions in various disease states (9, 10). RGMa has a peculiar influence on inflammation regulation in that it seems to have opposing functions during inflammation in different tissues (11). RGMa is expressed by macrophages at a level regulated by inflammatory stimuli (12). Analysis of single-nucleotide polymorphisms (SNPs) in patients with multiple sclerosis (MS) revealed associations between several functional loci in the *RGMa* promoter and the levels of cerebrospinal fluid interferon γ (IFN-γ) (13), a cytokine that plays an essential role in AS progression. It is postulated that certain functional polymorphisms in the *RGMa* promoter may be associated with inflammatory processes in some diseases. Our previous studies found that RGMa elicited negative effects on angiogenesis and inflammation development in ischemic brain injury (14–16). However, no previous studies have reported an association between RGMa and AS pathogenesis.

Recent studies have reported correlations between peripheral blood levels of netrin-1, Sema3E, and other NGPs and the degree of AS progression in adults (17, 18). Polymorphisms associated with increased serum Sema3E levels may affect carotid AS formation under the background of metabolic syndrome (19). Increasing evidence supports the use of NGPs as biomarkers for AS progression. Therefore, the availability of a functional SNP analysis on the genes encoding NGPs would be welcomed in the field of translational medicine (20). In the present study, we evaluated whether functional SNPs in the *RGMa* promoter were associated with AS burden in the cerebral arteries of patients with acute ischemic cerebrovascular accident.

## Materials and Methods

### Study Population

We designed a genetic association study by consecutively recruiting patients with first-ever acute ischemic cerebrovascular events. All participants were within 48 h of event onset and had a prior diagnosis of acute ischemic stroke or transient ischemic attack (TIA) upon admission to The First Affiliated Hospital of Chongqing Medical University from June 2019 to November 2020. Cerebral hemorrhage was excluded by computerized tomography scans. All participants were recruited from the Chinese Han population and were over 18 years of age. The exclusion criteria included: (1) a previous history of acute ischemic cerebrovascular accident; (2) a history of malignant tumor; (3) a history of autoimmune disease or chronic inflammatory disease; and (4) a diagnosis of cardioembolic stroke, stroke of other determined etiology, or a stroke of undetermined etiology based on TOAST classification (21).

The diagnosis was further reviewed after discharge. Participants with a final diagnosis of ischemic stroke or TIA and lacking the exclusion criteria listed above were included in subsequent evaluations. This study was approved by the Ethics Committee of The First Affiliated Hospital of Chongqing Medical University, and written informed consent was obtained from each recruited patient.

### Clinical Data Collection

After recruitment, we preliminarily enrolled 231 patients into this study. Under review, 19 patients were excluded in accordance with the exclusion criteria, and 11 with incomplete clinical data were excluded. The remaining 201 patients were included in the final analysis.

To evaluate intracranial and extracranial vascular AS burden, computerized tomography angiography (CTA) of the head and neck was conducted with a multi-slice scanner (GE Discovery CT750HD; GE Healthcare, Chicago, IL). The scanning parameters were 100 kV, 120 mAs, 5-mm section thickness, and intravenous administration of 80 mL non-ionic contrast (rate, 5 mL/s). Reconstructed time-of-flight magnetic resonance angiography was selected if CTA was contraindicated. The extent of cerebrovascular AS-related stenosis was examined in the intracranial artery (bilateral anterior, middle, and posterior cerebral arteries; basilar artery, and intracranial portions of the internal carotid and vertebral arteries) and extracranial artery (extracranial portions of the internal carotid and vertebral arteries). Two experienced neurologists who were blinded to the clinical information independently assessed all images. Final decisions were reached by a consensus. Quantifying CAB was determined in accordance with the extent of the stenosis, as previously described (22). Stenosis scores were recorded as 0 for no stenosis, 1 for <50% stenosis, 2 for 50–74% stenosis, 3 for >75% stenosis, or 4 for occlusion. The sum of cerebral AS scores was calculated and categorized into quartiles, and advanced CAB was defined to include patients exhibiting the highest quartile range of the AS scores.

Pro-atherogenic risk factors, which included total cholesterol, total triglycerides, low-density lipoprotein cholesterol (LDL-c), high-sensitivity C-reactive protein (hs-CRP), uric acid (UA), white blood cell count, and platelet count, were included. The estimated glomerular filtration rate (eGFR) was calculated using the Modification of Diet in Renal Disease formula [175 × (serum creatinine)^−1.154^ × (Age)^−0.203^ × (0.742 if female)]. An hs-CRP level > 3.0 mg/L was defined as a high vascular risk. Histories of hypertension, diabetes mellitus (DM), and smoking status were obtained for further multivariable regression analysis.

### Bioinformatic Analysis

We chose 3,000-bp upstream of the *RGMa* transcriptional start site as the promoter sequence region. FuncPred (https://snpinfo.niehs.nih.gov/snpinfo/snpfunc.html) was used to predict functional SNPs, and loci with altered transcription factor binding were identified (prediction results are shown in Supplementary Table 1) (23). From these results, we chose loci with minor allele frequencies > 0.05 in the Southern Chinese Han population from the 1,000 Genomes database (http://internationalgenome.org), and referred to previous related studies (13). Pairwise linkage disequilibrium was determined using the Linkage Disequilibrium Calculator to identify complete linkage disequilibrium in the specific population of the 1,000 Genome database in Ensembl (http://ensembl.org) (results are shown in Supplementary Table 2) (24). Three SNPs [rs4778099(G>A), rs10520720(G>A), and rs725458(C>T)] were selected for further analysis. Annotated SNP information was obtained from the dbSNP database (https://www.ncbi.nlm.nih.gov/snp/). Notably, the frequency of the minor allele T in rs725458 (66%), as annotated in the dbSNP, was higher than that of the major allele C (34%) in the Chinese Han population (1,000 Genome database). Tissue-specific expression quantitative trait loci (eQTL) information was obtained from the GTEx portal (http://www.gtexportal.org) and Blood eQTL Browser (https://genenetwork.nl/bloodeqtlbrowser/) (25, 26).

### Genotyping

Samples (5 ml) of EDTA-anticoagulated blood were collected from all recruits, and whole blood genomic DNA was isolated using a blood DNA isolation kit (TIANGEN, Beijing, China). Kompetitive allele-specific PCR (KASP) technology was used to genotype the selected SNPs. KASP master mix and primers were supplied by LGC Bioresearch Technology (Middlesex, UK), and KASP reactions were run on a compatible real-time PCR instrument (CFX Connect; Bio-Rad, Hercules, CA). Five percent of all genomic DNA samples, which were randomly selected for quality control, were Sanger sequenced.

### Gene Expression Omnibus (GEO) Data Retrieval

AS-related microarray dataset searches were conducted manually on NCBI's GEO database. Datasets containing artery samples or peripheral blood samples in independent cohorts were collected. For the RGMa differential expression analysis, the collected datasets with matched normal controls were selected (GSE7074, GSE23746, GSE20129, GSE57691). The RGMa expression array data in the AS group and normal control group were used in this analysis. Data in GSE7074 and GSE23746 datasets were obtained from independent carotid AS cohorts and their matched controls. Data in GSE20129 were obtained from an independent coronary artery AS cohort and matched controls. Data in GSE57691 were obtained from an independent aorta AS cohort and matched controls. For the AS plaque RGMa-related correlation analysis, only the datasets where the size of the target sample was >30 were selected (GSE125771, GSE24495), and the expression array data for RGMa, platelet-derived growth factor subunit B(PDGFB), actin alpha 2(ACTA2), and phosphatase and tensin homolog (PTEN) were used for further analysis. GSE125771 contained 40 independent AS carotid samples. GSE24495 contained 113 independent AS carotid samples.

### Statistical Analysis

SPSS (v26.0; IBM, Armonk, NY) was used for statistical analysis. We expressed continuous variables as the mean ± standard deviation (SD), and categorical values as frequencies (percentages). Student's *t*-tests and chi-square tests were performed for continuous values and categorical values, respectively. Hardy-Weinberg equilibrium analysis and the chi-square tests were performed for each locus. To evaluate the contribution of genotypes to CAB, multivariable logistics regression analysis was adjusted for common vascular risks (Model 1: age, sex, hypertension, DM, and smoking; Model 2: Model 1 plus eGFR, UA, LDL-c, and hs-CRP level). Haplotype analysis (rs4778099-rs10520720-rs725458) was performed using SNPstats (http://snpstats.net) (27). Our analysis of the association between haplotype and CAB was adjusted for age, sex, hypertension, smoking, eGFR, and hs-CRP level. GEO data correlation analysis was performed using Spearman's rank test. *P*-values of <0.05 were considered statistically significant. All tests were two-tailed.

## Results

### Patients' Characteristics

All enrolled patients were grouped according to their calculated CAB, and their baseline clinical data are shown in Table 1. Of the 201 enrolled patients, 55 were in the advanced CAB group and 146 were not considered to have advanced CAB. No significant differences among baseline clinical characteristics were found between the two groups, with the exception of the hs-CRP level (>3.0 mg/L), a well-known clinical marker for cerebral AS (28). The advanced CAB group contained a significantly higher proportion of patients with abnormal hs-CRP levels [Advanced CAB+: 29 (52.7%) vs. Advanced CAB–: 36 (24.7%), *p* < 0.001].

**Table 1:** Baseline clinical data distribution according to cerebral atherosclerosis burden.

**Characteristic**	**Advanced CAB** **(+)** **(*n* = 55)**	**Advanced CAB** **(–)** **(*n* = 146)**	***P*** **-value**
Age	64.0 ± 10.2	61.2 ± 10.7	0.094
Sex (male)	35 (63.6%)	104 (71.2)	0.299
Smoking	29 (52.7)	82 (56.2)	0.662
eGFR (mL/min/1.73 m^2^)	92.9 ± 24.5	94.2 ± 24.8	0.746
Hypertension	43 (78.2)	100 (68.5)	0.177
Diabetes mellitus	20 (36.4)	53 (36.3)	0.993
WBC (×10^9^/L)	7.7 ± 2.3	7.4 ± 2.5	0.504
PLT (×10^9^/L)	210.2 ± 58.3	209.7 ± 73.9	0.964
UA (μmol/L)	330.1 ± 79.5	354.2 ± 98.1	0.104
TG (mmol/L)	2.1 ± 1.9	2.0 ± 1.7	0.804
TC (mmol/L)	4.6 ± 1.6	4.6 ± 1.0	0.920
LDL-c (mmol/L)	2.9 ± 0.9	2.9 ± 0.9	0.782
hs-CRP level (>3.0 mg/L)	29 (52.7)	36 (24.7)	**<0.001**

*Data are presented as mean ± SD or n (%).*

*CAB, cerebral atherosclerosis burden; eGFR, estimated glomerular filtration rate; hs-CRP, high-sensitivity C-reactive protein; WBC, white blood cell counts; PLT, Platelet counts; UA, uric acid; TG, total triglyceride; TC, total cholesterol; LDL-c, low-density lipoprotein cholesterol. Statistically significant differences are shown in bold*.

Table 2 shows the allele and genotype frequencies of the target polymorphisms from our research. We also compared the genetic frequency between the enrolled patients and the Han population in Southern China (CHS), a population of age and sex matched healthy subjects published by the 1,000 Genome project. We found no significant difference in the target polymorphisms' allele and genotype frequency.

**Table 2:** Allelic and genotypic frequencies of the target *RGMa* promoter SNPs in enrolled patient group and healthy subject group.

	**CHS from 1,000 genome** **(*n* = 108)**	**Enrolled patients group** **(*n* = 201)**	***p*** **-value**
**rs4778099 (allele and genotype frequency)**			
A	70 (32.4%)	131 (32.6%)	0.964
G	146 (67.6%)	271 (67.4)	
AA	10 (9.3%)	23 (11.4%)	0.732
AG	50 (46.3%)	85 (42.3%)	
GG	48 (44.4%)	93 (46.3%)	
**rs10520720 (allele and genotype frequency)**			
A	34 (15.7%)	63 (15.7%)	0.982
G	182 (84.3%)	339 (84.3%)	
AA	2 (1.8%)	4 (2.0%)	0.994
AG	30 (27.8%)	55 (27.4%)	
GG	76 (70.4%)	142 (70.6%)	
**rs725458 (allele and genotype frequency)**			
T	135 (62.5%)	247 (61.4%)	0.796
C	81 (37.5%)	155 (38.6%)	
TT	43 (39.8%)	77 (38.3%)	0.965
TC	49 (45.4%)	93 (46.3%)	
CC	16 (14.8%)	31 (15.4%)	

*CHS, Han population in Southern China*.

### The RGMa Expression Pattern in Human Atherosclerotic Plaques and AS Are Correlated

**Figure 1:**
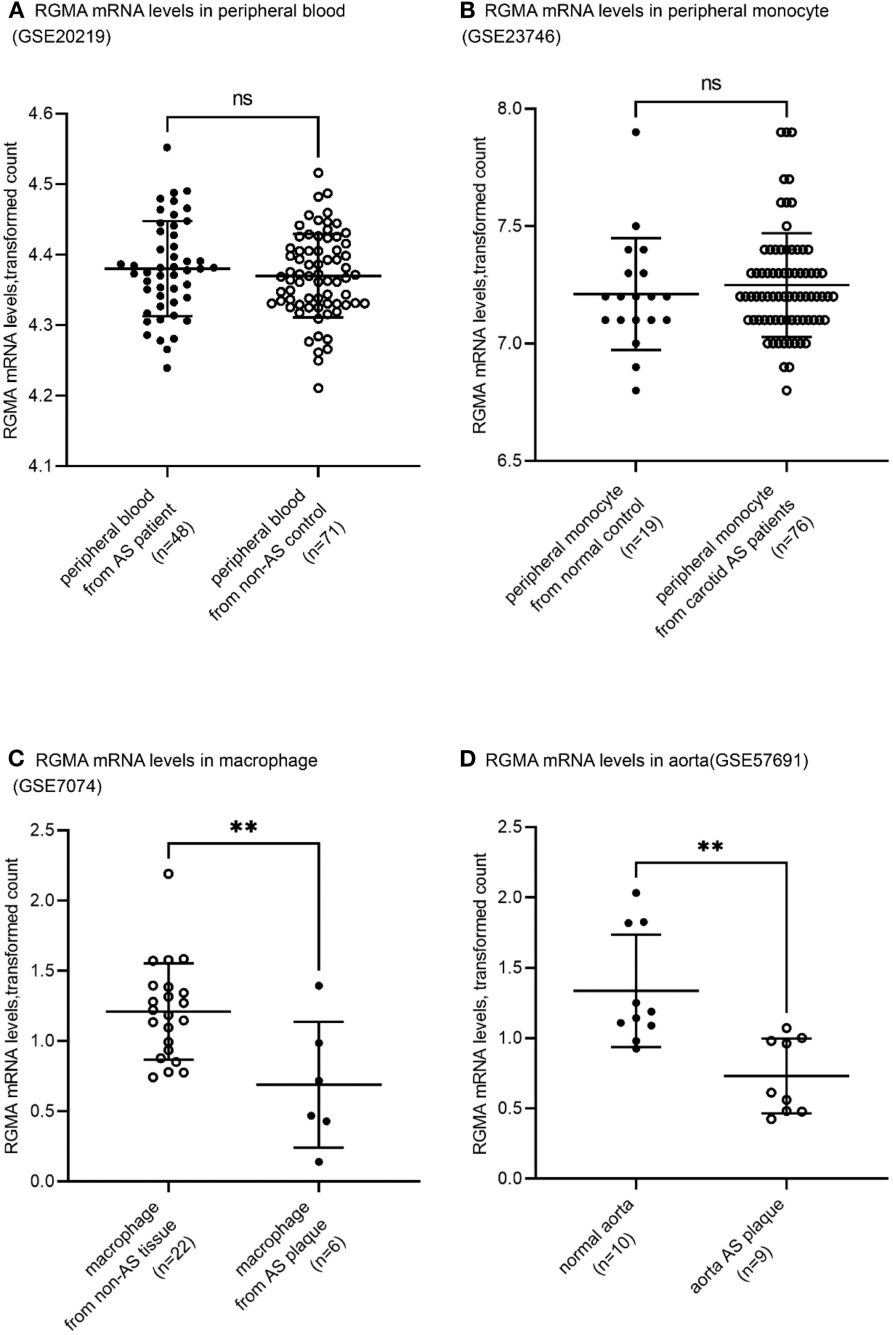
Tissue-specific RGMA expression pattern related to human atherosclerosis development. **(A,B)** The RGMA mRNA transcription level in peripheral blood and peripheral monocyte showed no significant discrepancy between AS-related patients and normal control. All data were derived from independent cohorts in GEO database (GSE20129, GSE23746). **(C,D)** The RGMA mRNA transcription levels in atheroma plaque and macrophages isolated from plaque were significantly higher than those in the normal artery and macrophage from non-AS tissue. All data were derived from independent cohorts in GEO database (GSE7074, GSE57691). There were 254 genes presenting the equal or higher differential expression magnitude than RGMa's in GSE7074. There were 2,122 genes presenting the equal or higher differential expression magnitude than RGMa's in GSE57691. The central horizontal lines indicate mean, and the upper and lower whisker marks indicate SD. ns, not significant; ^**^p < 0.01.

**Figure 2:**
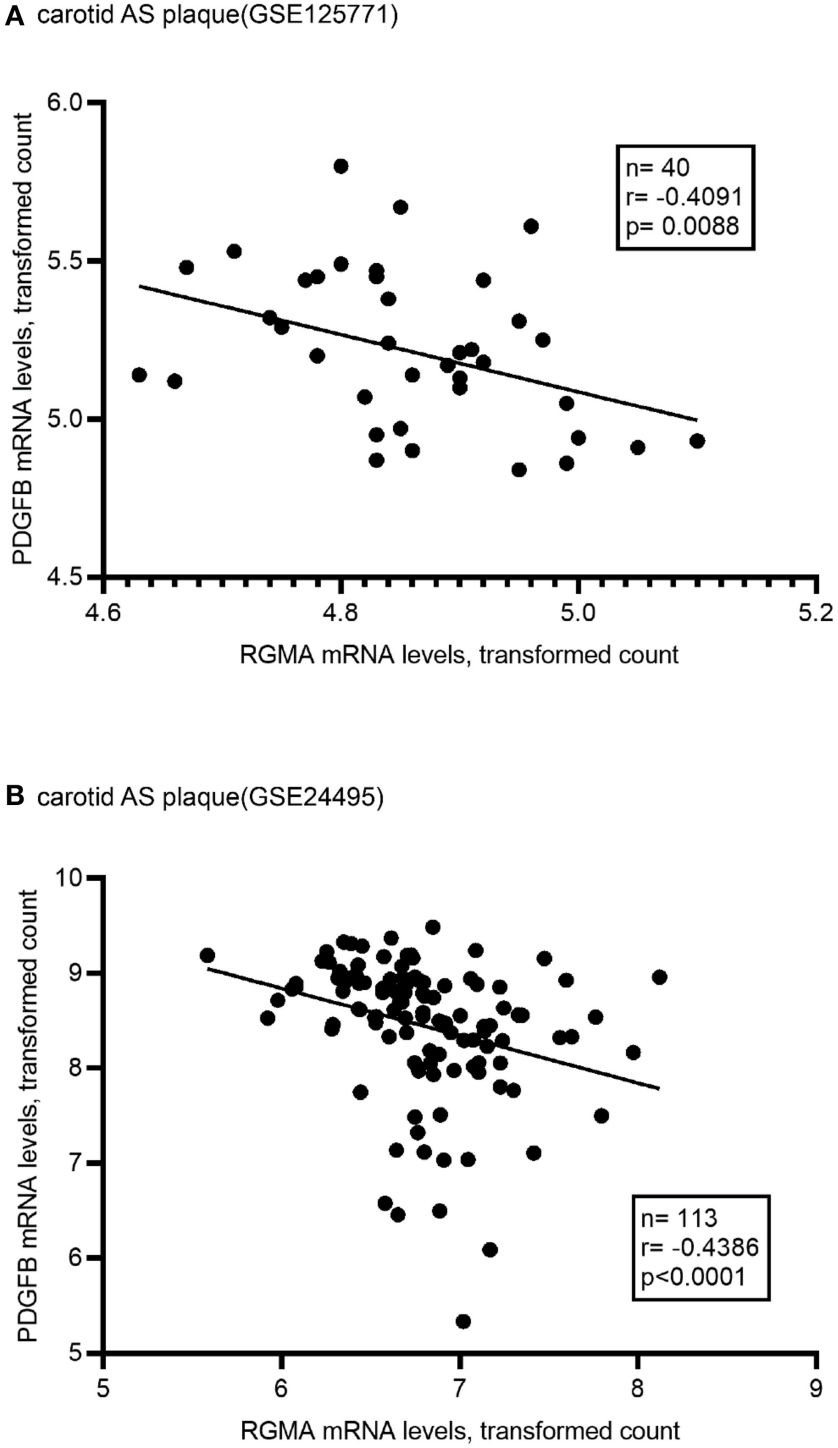
The association of RGMA with PDGFB in human carotid AS plaque. **(A,B)** In the two independent datasets from GEO database (GSE125771, GSE24495), RGMA mRNA transcription levels in carotid AS plaque negatively correlated with PDGFB mRNA levels. The correlation was tested by Spearman's rank test (**A**: *r* = −0.4091, *P* = 0.0088; **B**: *r* = −0.4386, *p* < 0.0001). The solid line indicates the linear regression fit of the measured data.

**Figure 3:**
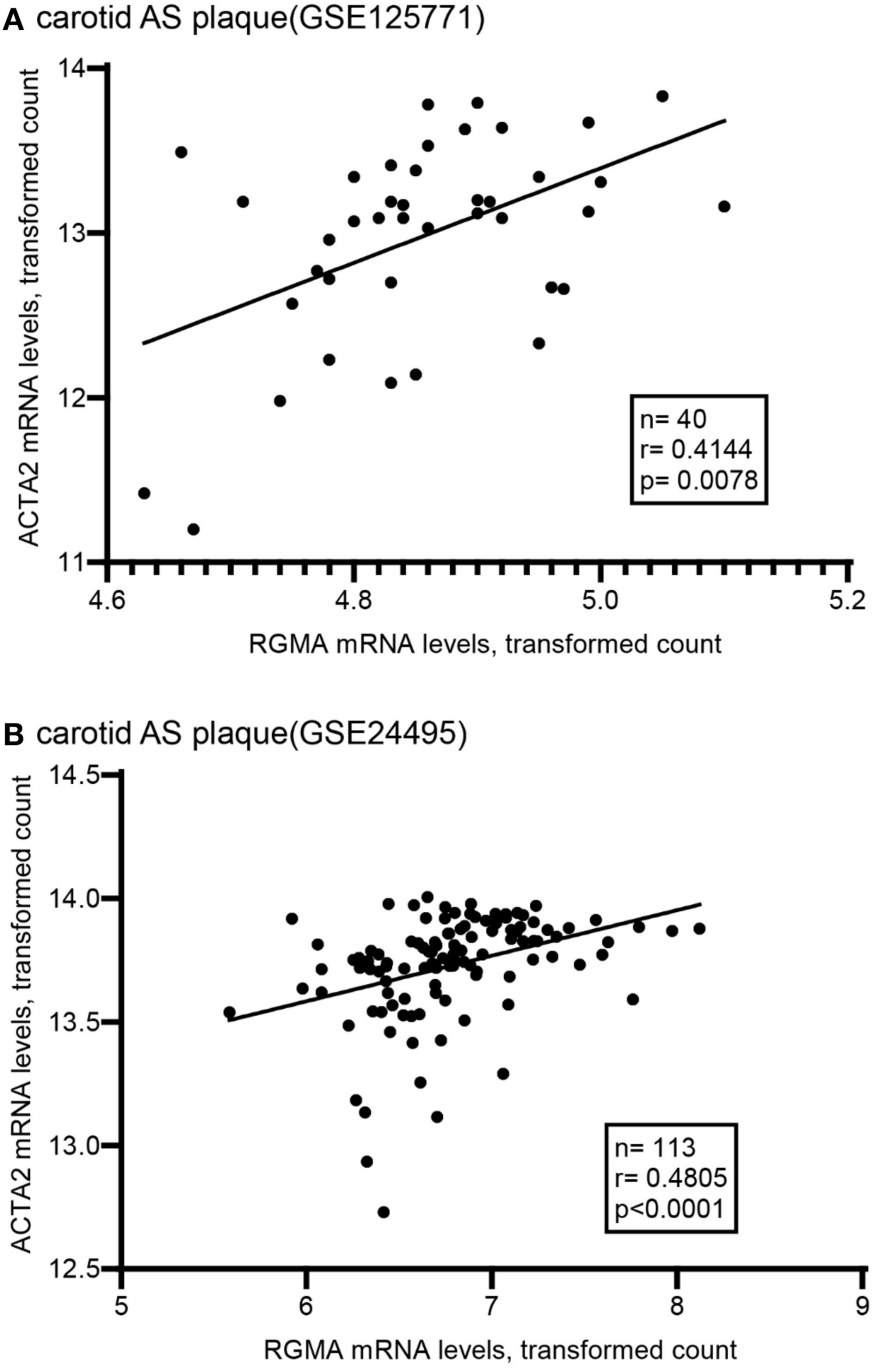
The association of RGMA with ATCA2 in human carotid AS plaque. **(A,B)** RGMA mRNA transcription levels in carotid AS plaque positively correlated with ATCA2 mRNA levels in the two independent datasets from GEO database (**A** according to GSE125771, **B** according to GSE24495). The correlation was tested by spearman's rank test (**A**: *r* = 0.4144, *P* = 0.0078; **B**: *r* = 0.4805, *p* < 0.0001).

**Figure 4:**
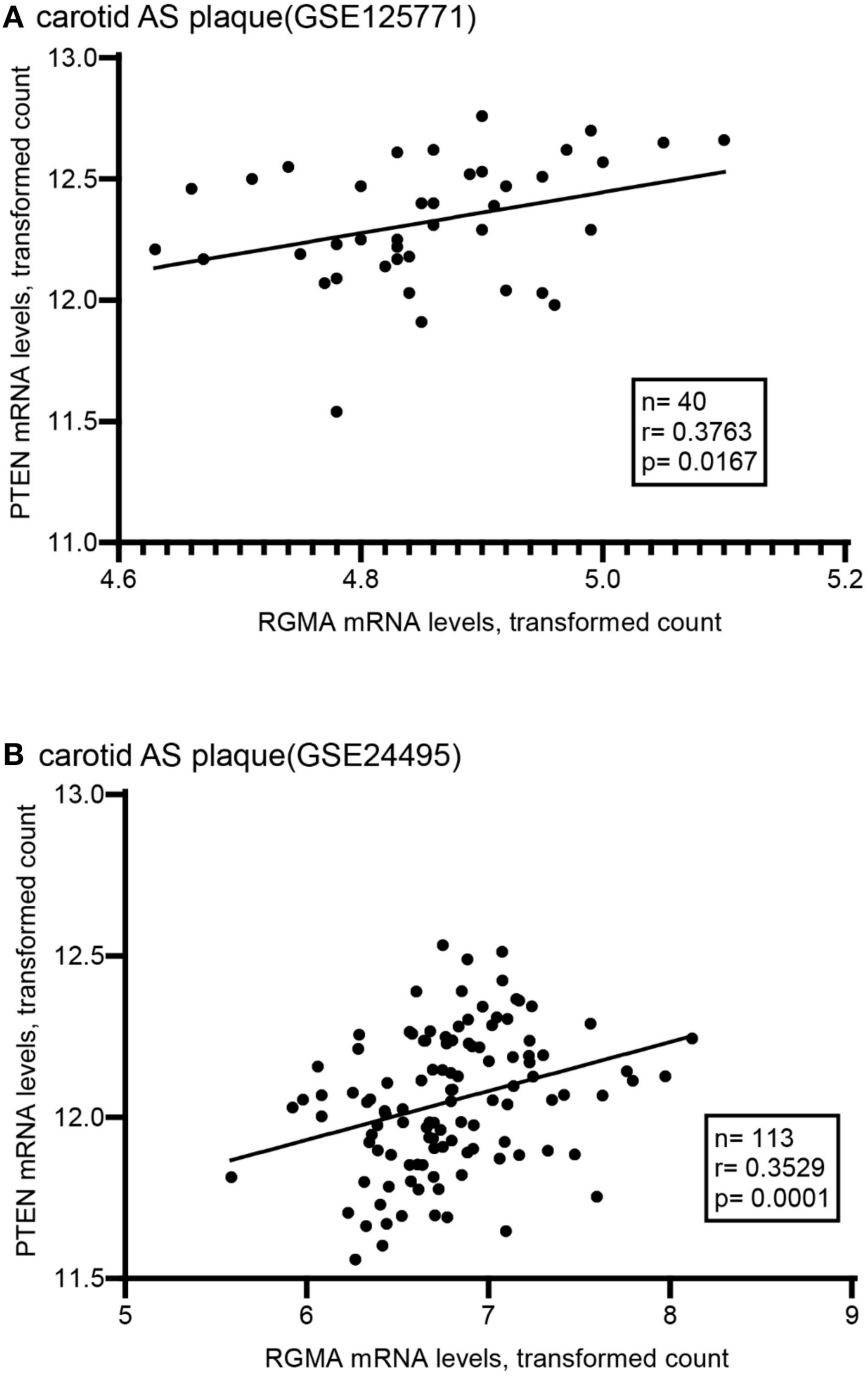
The association of RGMA with PTEN in human carotid AS plaque. **(A,B)** RGMA mRNA transcription levels in carotid AS plaque positively correlated with PTEN mRNA levels in the two independent datasets from GEO database (**A** according to GSE125771, **B** according to GSE24495). The correlation was tested by spearman's rank test (**A**: *r* = 0.3763, *p* = 0.0167; **B**: *r* = 0.3529, *p* = 0.0001).

Using the public GEO database, we analyzed the RGMa expression patterns in various tissues and cells between AS and non-AS states. Data from GSE20129 and GSE23746 indicated no significant difference in the RGMa levels in peripheral blood or peripheral monocytes between patients with AS and non-AS controls. Nevertheless, RGMa levels in atherosclerotic aortas or in macrophages isolated from plaques were much lower than those in normal aortas or macrophages from normal tissue (Figure 1). We also investigated whether RGMa levels were correlated with AS-related molecule expression. We found a negative correlation between RGMa levels and *PDGFB*, a crucial pro-atherogenic gene (Figure 2). RGMa levels were also positively correlated with the transcription levels of *ACTA2* and *PTEN*, which are regarded as markers of normal quiescent vascular smooth muscle cells and vascular protective factor, respectively (Figures 3, 4) (29, 30).

### Associations Between RGMa Promoter SNPs and Haplotypes With CAB

We investigated whether associations between target SNPs and CAB were present in the patients. Table 3 shows the frequency of all SNPs in each group. Compliance with Hardy-Weinberg equilibrium (*p* > 0.05) was tested. Logistic regression models were used in accordance with additive, recessive and dominant inheritance models in this analysis. We found the rs725458 T allele (in the additive or dominant models) was independently associated with increased CAB after adjusting for common vascular risk factors (adjusted OR = 1.66, *P* = 0.046; adjusted OR = 7.18, *P* = 0.010, respectively). The rs4778099 AA genotype was inversely associated with CAB in the recessive model (adjusted OR = 0.10, *P* = 0.027). rs10520720 was not significantly associated with CAB in this analysis.

**Table 3:** Association between predicted functional SNP in RGMA promoter and cerebral atherosclerosis burden adjusted for common vascular risk factors.

	**Genotype**	**Advanced CAB (+)** **(*n* = 55)**	**Advanced CAB (–)** **(*n* = 146)**	**Adjusted model 1**		**Adjusted model 2**	
				**aOR (95% CI)**	***P*** **-value**	**aOR (95% CI)**	***P*** **-value**
rs4778099							
Additive:	GG	27 (49.1%)	58 (39.7%)	0.60 (0.36–1.00)	**0.049**	0.62 (0.37–1.06)	0.077
	GA	27 (49.1%)	66 (45.2%)				
	AA	1 (1.8%)	22 (15.1%)				
Dominant:	GG	27 (49.1%)	58 (39.7%)	0.71 (0.37–1.35)	0.299	0.78 (0.40–1.54)	0.472
	GA+AA	28 (50.9%)	88 (60.3%)				
Recessive:	AA	1 (1.8%)	22 (15.1%)	0.11 (0.01–0.83)	**0.033**	0.10 (0.01–0.77)	**0.027**
	GA+GG	54 (98.2%)	124 (84.9%)				
rs10520720							
Additive	GG	36 (65.5%)	106 (72.6%)	1.45 (0.78–2.69)	0.236	1.77 (0.91–3.44)	0.092
	GA	17 (30.9%)	38 (26.0%)				
	AA	2 (3.6%)	2 (1.4%)				
Dominant:	AA+AG	19 (34.5%)	40 (27.4%)	1.40 (0.70–2.78)	0.344	1.71 (0.82–3.56)	0.154
	GG	36 (65.5%)	106 (72.6%)				
Recessive:	AA	2 (3.6%)	2 (1.4%)	3.28 (0.43–25.36)	0.255	4.56 (0.53–39.63)	0.169
	AG+GG	53 (93.4%)	144 (98.6%)				
rs725458							
Additive:	TT	25 (45.5%)	52 (35.6%)	1.78 (1.08–2.91)	**0.023**	1.66 (1.01–2.74)	**0.046**
	TC	28 (50.9%)	65 (44.5%)				
	CC	2 (3.6%)	29 (19.9%)				
Dominant:	TT+TC	53 (93.4%)	117 (80.1%)	6.35 (1.45–27.89)	**0.014**	7.18 (1.60–32.30)	**0.010**
	CC	2 (3.6%)	29 (19.9%)				
Recessive:	TT	25 (45.5%)	52 (35.6%)	1.52 (0.80–2.91)	0.206	1.30 (0.66–2.57)	0.450
	TC+CC	30 (54.5%)	94 (64.4%)				

*Model 1: Adjusted for age, sex, hypertension, Diabetes mellitus, smoking.*

*Model 2: Adjusted for Model1 plus hsCRP level (>3.0), UA, eGFR, LDL-c.*

*aOR, adjusted odds ratio. Statistically significant differences are shown in bold*.

**Figure 5:**
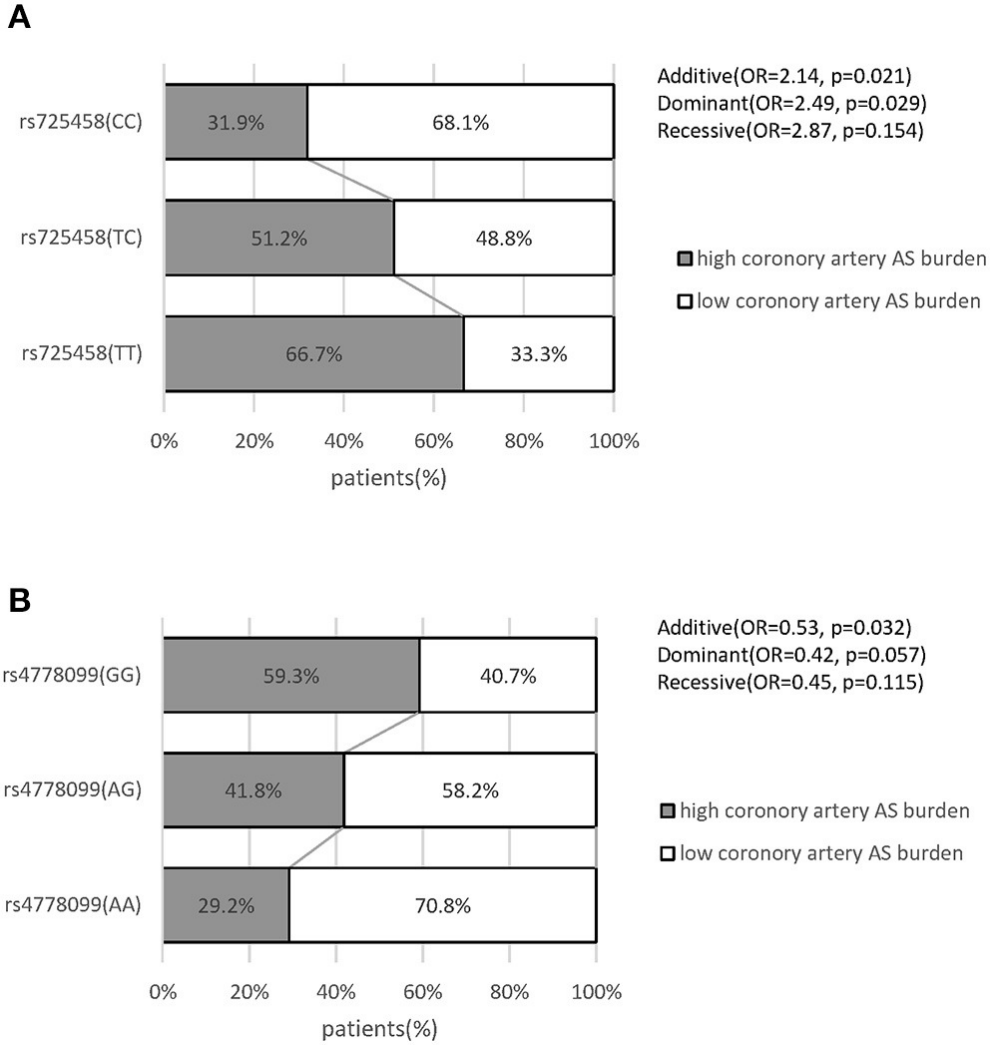
Distribution of coronary artery atherosclerosis burden in patients grouped by genotype and the association with target SNPs in GSE90073. **(A)** Grouped by rs725458 genotype. **(B)** Grouped by rs4778099 genotype. All data were collected from the publicly available GSE90073 dataset in GEO database. *P*-value and OR were calculated by logistic regression analysis. AS, atherosclerosis.

To further substantiate our results, we queried the GEO database to verify the association between our target SNPs and atherosclerosis from publicly available SNP-array data in related studies. Data in GSE90073 were collected from an independent cohort of 106 American patients (African ancestry, 24; European ancestry, 82) who were undergoing clinically indicated diagnostic cardiac catheterization (Supplementary Table 3). Coronary artery atherosclerosis burden was quantified by an ordinal coronary artery disease score (range 0–4) according to the angiographic stenosis distribution (31). The patient's genome-wide SNP identification was obtained via an SNP array. Complete genotyping data and atherosclerosis quantification data were in public access. In this analysis, we found a similar genotype-atherosclerosis burden distribution (high atherosclerosis burden is defined as coronary artery disease score equal to 3 or 4) with our study (Figure 5). We also tested the association in the logistic regression model. It supported the rs725458 T allele (in the additive or dominant models) was associated with high atherosclerosis burden (OR = 2.14, *P* = 0.021; OR = 2.49, *P* = 0.029, respectively) and the rs4778099 A allele was inversely associated with atherosclerosis burden in the additive model (OR = 0.53, *P* = 0.032). These findings suggested the consistent effect of our target SNPs on atherosclerosis across ancestries.

The estimated haplotype distribution from our analysis of the selected SNPs is shown in Table 4. GGT was the most frequent haplotype in both groups (55.7 and 43.5%, respectively). In the haplotype association analysis, no haplotype exhibited a significant association with CAB after adjustment.

**Table 4:** Haplotype-based association analysis of cerebral atherosclerosis burden.

**Haplotype (rs4778099-rs10520720-rs725458)**	**Frequency**		**Unadjusted model**		**Adjusted model**	
	**Advanced CAB (+)**	**Advanced CAB** **(–)**	**OR** **(95% CI)**	***P*** **-value**	**aOR** **(95%CI)**	***P*** **-value**
GGT	0.557	0.435	1.00 (ref)		1.00 (ref)	
AGC	0.204	0.377	0.55 (0.32–0.93)	**0.028**	0.57 (0.30–1.08)	0.088
GAT	0.133	0.144	1.06 (0.56–2.01)	0.860	1.04 (0.42–2.55)	0.930
GGC	0.036	0.045	0.64 (0.18–2.31)	0.490	0.62 (0.16–2.45)	0.500

*Adjusted for age, sex, HP, smoking, eGFR, hs-CRP level (>3.0). Statistically significant differences are shown in bold*.

## Discussion

In this exploratory study, we focused on identifying potential associations between *RGMa* transcription levels and the degree of cerebrovascular AS, while also investigating whether any functional SNP in the *RGMa* promoter region affected AS progression in a specific group of patients with acute ischemic cerebrovascular events. We found that *RGMa* mRNA levels correlated with human atherosclerotic plaque development in GEO analysis and several SNPs (rs725458 and rs4778099) in the *RGMa* promoter were associated with CAB in patients diagnosed with acute ischemic cerebrovascular accident in the Chinese Han population. We also used haplotype analysis based on functional SNPs to improve the accuracy of genetic association prediction. However, it did not lead to a better prediction. We believed that a relatively low SNP number in the haplotype and inadequate linkage disequilibrium within the block might hamper the prediction (32).

RGMa is a promising target for the clinical diagnosis and treatment of numerous diseases based on its versatility, as verified by abundant preclinical or clinical observational studies (33–36). However, our study is the first to investigate the potential relationship between RGMa and AS. The negative correlation we identified between *RGMa* transcript levels and a crucial cerebrovascular stenosis risk gene, PDGFB (37), along with a discrepancy in *RGMa* transcription between normal arteries and atheroma plaques, suggests potential vasoprotective effects for RGMa. RGMa, which was found to be expressed in several types of cells in vessel walls and in the immune system, plays a role in AS development (12, 38). However, animal models or cell-based assays are still needed to further confirm this.

RGMa-related translational medical research activity has recently increased, especially with regard to MS (35) and peritonitis (39). Indeed, exploring RGMA polymorphisms has become one of the favored research directions (13, 40, 41). In an eQTL study of MS-related inflammatory cytokine genes, Nohra et al. identified associations between SNPs in *RGMa* and MS morbidity in Nordic males. Several *RGMa* SNPs were also correlated with IFN-γ and interleukin 6 levels in the cerebrospinal fluids from MS patients, and most (rs6497019, rs10520720, and rs725458) were concentrated within the promoter or enhancer regions of *RGMa* (13). Although that study did not discuss the function of these polymorphisms, it is understood today that *RGMa* expression affects local inflammatory states and cytokine levels in a diverse range of diseases (42, 43). We postulate that these functional SNPs or eQTLs in the *RGMa* promoter region are associated with *RGMa* transcription in specific conditions, thereby affecting inflammatory regulation. As was found in our research, patients with the rs725458 T allele presented with higher CAB than those with the C allele. Functional SNPs in promoter region usually affect gene expression by altering regulatory motifs. We queried for any regulatory motif altered by our target SNPs in HaploReg (https://pubs.broadinstitute.org/mammals/haploreg/haploreg.php) and JASPAR (http://jaspar.genereg.net) database, and found that: the variant in rs725458 could significantly influence the bind of CEBPB (CCAAT/Enhancer Binding Protein Beta) and CEBPD (CCAAT Enhancer Binding Protein Delta); the variant in rs4778099 could impact the binding of Hoxc10 (Homeobox C10). CEBPB and CEBPD are both common transcription factors participating inflammatory regulation process (44, 45). Studies reported that they both affect atherosclerosis progression (46, 47). No atherosclerosis-related study was found in our query for Hoxc10, and no study has reported any relation between these three factors and RGMa. Our future work will focus on whether the target SNPs influence *RGMa* transcription through altered binding between these transcription factors and loci.

One study reported that an *RGMa* trans-eQTL in the human frontal cortex (rs12442183 T allele) is related to increased opioid dependence. Experiments with animal models have also indicated that morphine administration upregulates RGMa expression in the striatum (40). Hence, it is believed that eQTL analysis of RGMa should provide meaningful data for clinical translational studies. We did not identify any cerebral artery-specific or aorta-specific *RGMa* eQTLs in the GTEx portal. However, the query for *RGMa* in the Blood eQTL Browser implicated rs4778099, rs10520720, and rs725458 in *RGMa* cis-eQTLs for human peripheral blood (FDR < 0.1). Although RGMa transcription levels in peripheral blood and peripheral monocytes did not appear to be significantly discrepant between patients with AS and normal controls, we observed lower RGMa levels in macrophages isolated from atheroma plaques when compared with other normal tissues.

Bone marrow-derived macrophages are the primary inflammatory cell type involved in AS formation, and M1/M2 macrophage differentiation is a key point in AS inflammation regulation (48, 49). RGMa expression levels in macrophages decrease as they change from an M2 to an M1 phenotype, and this is coupled with a series of pro-inflammatory reactions. Increased exogenous RGMa levels were also found to improve M2 polarization of macrophages and inhibit monocyte recruitment to inflammatory sites (12, 50). Therefore, based on the analyses described above and our own results, we believe that these inflammation-related SNPs in the *RGMa* promoter possibly affect CAB in specific patient groups, although the underlying mechanism needs further experimental validation.

There were several limitations in this study. First, although this was a consecutive enrollment study, it was a single-center trial and the low sample size of the recruited patients limited its statistical power. However, we used the Genetic Power Calculator (http://zzz.bwh.harvard.edu/gpc/cc2.html) to estimate the power, and the sample size could still achieve a power of over 0.8 with an alpha value of 0.05. Second, data for other vascular risk factors, such as body mass index and serum homocysteine, were not collected. These factors could affect the independence of the regression analysis. Third, we mainly discussed the stenosis of intracranial and extracranial arteries as representative of CAB. However, other aspects (e.g., plaque vulnerability and calcification) will still need to be investigated, and were beyond the range of data available to us.

## Conclusions

This study is the first to identify a relationship between *RGMa* and cerebrovascular AS, and our results suggest a potential vasoprotective function for *RGMa*. Functional SNPs in the *RGMa* promoter were found to be independently associated with CAB in patients with acute ischemic cerebrovascular accident. Collectively, these results offer a promising new direction for *RGMa*-related translational studies on AS.

## Data Availability Statement

The original contributions presented in the study are included in the article/Supplementary Material, further inquiries can be directed to the corresponding author.

## Ethics Statement

The studies involving human participants were reviewed and approved by the Ethics Committee of The First Affiliated Hospital of Chongqing Medical University. The patients/participants provided their written informed consent to participate in this study.

## Author Contributions

QH was responsible for study design, data collection and analysis, lab experiments, and manuscript drafting. ZC, XY, and SL were involved in data collection and analysis. RZ was involved in statistical analysis and lab experiments. XQ was involved in technical support and manuscript revision. All authors contributed to the article and approved the submitted version.

## Funding

This work has been supported by grants given by the National Natural Science Foundation of China (Grant Number: 82071338).

## Conflict of Interest

The authors declare that the research was conducted in the absence of any commercial or financial relationships that could be construed as a potential conflict of interest.

## Publisher's Note

All claims expressed in this article are solely those of the authors and do not necessarily represent those of their affiliated organizations, or those of the publisher, the editors and the reviewers. Any product that may be evaluated in this article, or claim that may be made by its manufacturer, is not guaranteed or endorsed by the publisher.

## Abbreviations

RGMa: repulsive guidance molecule a
CAB: cerebral atherosclerosis burden
SNP: single-nucleotide polymorphism
OR: odds ratio
CI: confidence interval
AS: atherosclerosis
NGP: neural guidance protein
Sema3E: Semaphorin 3E
MS: multiple sclerosis
IFN-γ: interferon γ
TIA: transient ischemic attack
CTA: computerized tomography angiography
LDL-c: low-density lipoprotein cholesterol
hs-CRP: high-sensitivity C-reactive protein
UA: uric acid
eGFR: estimated glomerular filtration rate
DM: diabetes mellitus
eQTL: expression quantitative trait loci
KASP: Kompetitive allele-specific PCR
GEO: Gene Expression Omnibus
PDGFβ: platelet-derived growth factor subunit B
ACTA2: actin alpha 2
PTEN: phosphatase and tensin homolog
SD: standard deviation
CHS: Han population in Southern China.
