# Supplementary material for: Common Polymorphisms in the RGMa Promoter Are Associated With Cerebrovascular Atherosclerosis Burden in Chinese Han Patients With Acute Ischemic Cerebrovascular Accident
Source: Front Cardiovasc Med. 2021 Oct 15;8:743868. doi: 10.3389/fcvm.2021.743868 (PMC8554026; doi:10.3389/fcvm.2021.743868)
Supplement: Supplementary file 1 [file Table_1.DOCX]

|  | Allele | Transcription factor binding site | Minor Allelic Frequencies of East Asian Population in ALFA | Minor Allelic Frequencies of Han Population in Southern China in 1000 Genome |
| --- | --- | --- | --- | --- |
| rs63695060 | T>C | √ | 0.00 | - |
| rs62045497 | A>G | √ | 0.00 | - |
| rs60048376 | C>G | √ | 0.00 | 0.00 |
| rs34933545 | G>A | √ | 0.00 | - |
| rs60448961 | G>A | √ | - | - |
| rs4778099 | G>A | √ | 0.19 | 0.33 |
| rs10520720 | G>A | √ | 0.19 | 0.15 |
| rs725458 | C>T | √ | 0.62 | 0.62 |
| rs59118360 | G>C | √ | - | - |
| rs11074140 | G>C | √ | 0.63 | 0.62 |
| rs28588884 | G>T | √ | 0.00 | 0.00 |

**Supplementary Table 1**. RGMa promoter SNPs’ function prediction analysis in FuncPred

Only SNPs in the predicted transcription factor binding sites were included.

Abbreviation: ALFA, Allele Frequency Aggregator, published in National Center for Biotechnology Information(NCBI).
