# Supplementary material for: Common Polymorphisms in the RGMa Promoter Are Associated With Cerebrovascular Atherosclerosis Burden in Chinese Han Patients With Acute Ischemic Cerebrovascular Accident
Source: Front Cardiovasc Med. 2021 Oct 15;8:743868. doi: 10.3389/fcvm.2021.743868 (PMC8554026; doi:10.3389/fcvm.2021.743868)
Supplement: Supplementary file 2 [file Table_2.DOCX]

**Supplementary Table 2**. Linkage Disequilibrium among functional SNPs(minor allele frequency > 0.05) calculated by Linkage Disequilibrium Calculator

| SNP pairs | r^2^ | D’ |
| --- | --- | --- |
| rs4778099-rs10520720 | 0.086573 | 0.999890 |
| rs10520720-rs11074140 | 0.106550 | 0.999884 |
| rs10520720-rs725458 | 0.106550 | 0.999884 |
| rs4778099-rs11074140 | 0.812498 | 0.999999 |
| rs4778099-rs725458 | 0.812498 | 0.999999 |
| rs725458-rs11074140 | 1.000000 | 1.000000 |

Linkage disequilibrium was calculated using genotype data from CHS population in 1000 Genome.

Analysis showed rs725458 and rs11074140 were in complete linkage disequilibrium. As rs725458 had been reported in other studies for its clinical significance, here we selected rs725458 for further association analysis.
