# Supplementary material for: Common Polymorphisms in the RGMa Promoter Are Associated With Cerebrovascular Atherosclerosis Burden in Chinese Han Patients With Acute Ischemic Cerebrovascular Accident
Source: Front Cardiovasc Med. 2021 Oct 15;8:743868. doi: 10.3389/fcvm.2021.743868 (PMC8554026; doi:10.3389/fcvm.2021.743868)
Supplement: Supplementary file 3 [file Table_3.DOCX]

**Supplementary Table 3**. Allelic and Genotypic Frequencies of rs4778099 and rs725458 in GSE90073 Grouped by Coronary Artery Atherosclerosis Burden

|  | High coronary artery atherosclerosis burden | Low coronary artery atherosclerosis burden | |
| --- | --- | --- | --- |
| rs4778099(allele and genotype frequency) | | |  |
| A | 37(40.2%) | 66(55.0%) | |
| G | 55(59.8%) | 54(45.0%) | |
| AA | 7(15.2%) | 17(28.3%) | |
| AG | 23(50.0%) | 32(53.3%) | |
| GG | 16(34.8%) | 11(18.3%) | |
| rs725458(allele and genotype frequency) | | |  |
| T | 34(39.5%) | 27(24.1%) | |
| C | 52(60.5%) | 85(75.9%) | |
| TT | 6(14.0%) | 3(5.4%) | |
| TC | 22(51.2%) | 21(37.5%) | |
| CC | 15(34.8%) | 32(57.1%) | |
